# Allogenic MSCs are a safe and efficacious treatment for knee osteoarthritis: A systematic review of randomised controlled trials

**DOI:** 10.1002/jeo2.70437

**Published:** 2025-09-18

**Authors:** Randy Y. J. Loke, Zachary Chu, Jonathan Liang, Don Koh, Junwei Soong, Kong Hwee Lee, Hamid Rahmatullah Bin Abd Razak

**Affiliations:** ^1^ Department of Orthopaedic Surgery Singapore General Hospital Singapore Singapore; ^2^ Yong Loo Lin School of Medicine National University of Singapore Singapore Singapore; ^3^ Total Orthopaedic Care & Surgery Pte Ltd Singapore Singapore; ^4^ SingHealth Duke‐NUS Musculoskeletal Sciences Academic Clinical Programme Singapore Singapore

**Keywords:** allografts, knee osteoarthritis, mesenchymal stem cells, platelet‐rich plasma

## Abstract

**Purpose:**

Implantation of mesenchymal stem cells (MSCs) is a potential non‐surgical option for cartilage repair. Currently, its clinical use largely focuses on focal cartilage defect repair and intra‐articular injections in knee osteoarthritis (OA). Most studies have looked at the efficacy of MSCs from autologous sources, which can be limited by inter‐patient variability of age, diseases, sites and methods of harvest. This systematic review aims to evaluate studies that focus on allogeneic MSCs implantation versus placebo in patients with knee OA to summarise the efficacy and safety of allogeneic MSCs in knee OA.

**Methods:**

A systematic search following PRISMA guidelines was performed in PubMed, Scopus and EMBASE. Original studies investigating the outcomes of allogeneic MSC implantations in patients with knee OA were included. Data on clinical outcomes, such as subjective scores such as VAS and WOMAC, radiological outcomes, such as cartilage thickness, and histological outcomes, such as ICRSII score, were extracted.

**Results:**

Seven studies were included in this review. There was 30.4 points improvement in Visual Analogue Scale scores and 40.0 points of improvement in Western Ontario and McMaster Universities Osteoarthritis Index scores at 12 months. Improved cartilage thickness and decreased poor cartilage quality as measured by T2 relaxation. Measurements at the lesion site were observed in three studies as assessed by postoperative magnetic resonance imaging and this was correlated clinically. One study also showed histological improvement with overall ICRSII scores significantly improving from 32.0 ± 21.6 at baseline to 55.9 ± 23.2 at 6 months (*p* < 0.05). No major complications or tumorigenesis occurred.

**Conclusion:**

Allogeneic MSC implantation in patients with knee osteoarthritis provides sustained clinical improvement and satisfactory cartilage restoration, up to 12 months follow‐up. These results are supported by both imaging and histological studies. The safety profile of allogeneic MSCs is excellent, with minimal adverse events mainly limited to local reaction to injection and no long‐term adverse effects.

**Level of Evidence:**

Level II.

**Abbreviations**
MSCs: mesenchymal stem cells
OA: osteoarthritis
PRP: platelet‐rich plasma
VAS: Visual Analogue Scale
WOMAC: Western Ontario and McMaster Universities Osteoarthritis Index

## INTRODUCTION

Knee osteoarthritis (OA) is a chronic, debilitating disease characterised by knee pain, swelling, and stiffness, predominantly affecting the elderly and obese populations. With approximately 13% of women and 10% of men aged 60 years and above in the United States experiencing symptomatic knee OA, the global incidence and burden of this ailment are expected to surge alongside ageing populations [33].

In addition to conventional non‐surgical management like exercise therapy and analgesics, orthobiologics have been gaining popularity as a minimally invasive mode of treatment [6]. These biological options are being explored for their potential to regenerate cartilage either through blood‐derived products such as autologous whole blood cells and platelet‐rich plasma (PRP) that stimulate angiogenesis and chondrocyte proliferation [1, 26] or through cellular products such as stem cells which modulate the tissue microenvironment via various mechanisms to stimulate regeneration [16].

Among the available orthobiologic options, mesenchymal stem cells (MSCs) have garnered significant attention [23]. Derived from sources such as bone marrow, adipose tissue, and synovial membrane [25], MSCs are multipotent progenitor cells capable of differentiating into chondrocytes, osteocytes, and stromal cells, making them promising candidates for cartilage regeneration in OA.

Autologous MSCs, although beneficial, pose challenges such as invasive harvesting procedures and variability in cell yield, particularly in older patients [17]. Allogenic MSCs on the other hand, can potentially bypass these issues to provide a convenient product with consistent quality, high plasticity, self‐renewing potential, and immunomodulatory properties [32]. Allogenic MSCs also do come with other considerations such as the sourcing of MSCs and donor selection [21, 27], its associated preservation and storage [2], and concerns of an autoimmune response [4]. Given recent advances in allogenic MSCs showing promising clinical outcomes in the context of knee OA without significant adverse events, this systematic review aims to evaluate the current literature on the safety and efficacy of allogenic MScs versus placebo in knee OA and to propose directions for future research.

## MATERIALS AND METHODS

This systematic review follows the Preferred Reporting Items for Systematic Reviews and Meta‐Analyses (PRISMA) guidelines.

### Research question

What is the efficacy of intra‐articular injection of allogenic MSCs in patients with knee OA compared to saline control on pain score and function?

### Search strategy and search eligibility criteria

A literature search was systematically conducted in PubMed (Medline), Scopus and EMBASE databases. The following search algorithm was used for all databases: (“mesenchymal stem cells” OR “MSC”) AND (“osteoarthritis” OR “OA” OR “degeneration” OR “gonarthrosis”) AND “knee”, with the search being limited to Title, Abstract and Keywords. The search was performed by two independent investigators (RL and ZC) and updated before the final analyses on 15/6/24. Computer de‐duplication was performed. A manual review of the references of selected articles was also completed to add studies that were originally missed.

The inclusion criteria are as follows: (i) Randomised controlled trials using allogeneic stem cells in patients with knee OA, with reported qualitative outcome measures (ii) studies published in the English language.

Exclusion criteria were as follows: (i)articles that used autologous MSCs to treat OA patients, (ii) reviews, conference submissions/abstracts or letters to the editor, (iii) preclinical in vitro or animal studies, and (iv) articles not published in English.

### Study selection

The search was performed by two independent reviewers (RL and ZC). Any discrepancies and disagreements were resolved by consensus. Relevant studies were identified via abstract screening and subsequent full‐text evaluation. References of the included studies were extracted and manually reviewed to further identify eligible studies, according to the snowball method. Investigators were blinded to each other during study selection and discrepancies were resolved by consensus, including the senior author (HR).

### Quality assessment

A list of criteria recommended by the Cochrane Collaboration was used to assess the studies. Oxford Centre for Evidence‐Based Medicine (OCEBM) level of evidence (LoE) was assigned to score each study. Since only randomised controlled trials were included, the studies were scored based on the modified Jadad scale [19]. This included domains of randomisation binding, account for withdrawals, inclusion/exclusion criteria, and method of statistical analysis. Two authors (ZC and JL) independently scored the studies, and discrepancies were resolved by consensus, including the senior author(HR).

### Data extraction

The authors independently extracted data from eligible studies according to a predefined data extraction form as per protocol. Data extracted includes study characteristics, patient demographics (sample size, age, gender), number of MSCs applied and clinical outcome measures. The primary outcome was the improvement in clinical score in the patients' knee OA.

### Statistical analysis

Mean and standard deviation were calculated for continuous variables while absolute and relative frequencies were reported for categorical variables. Statistical analysis and graphs were plotted using GraphPad Prism 8.0 for Windows (GraphPad Software, San Diego, CA, USA).

## RESULTS

### Study identification

**Figure 1:**
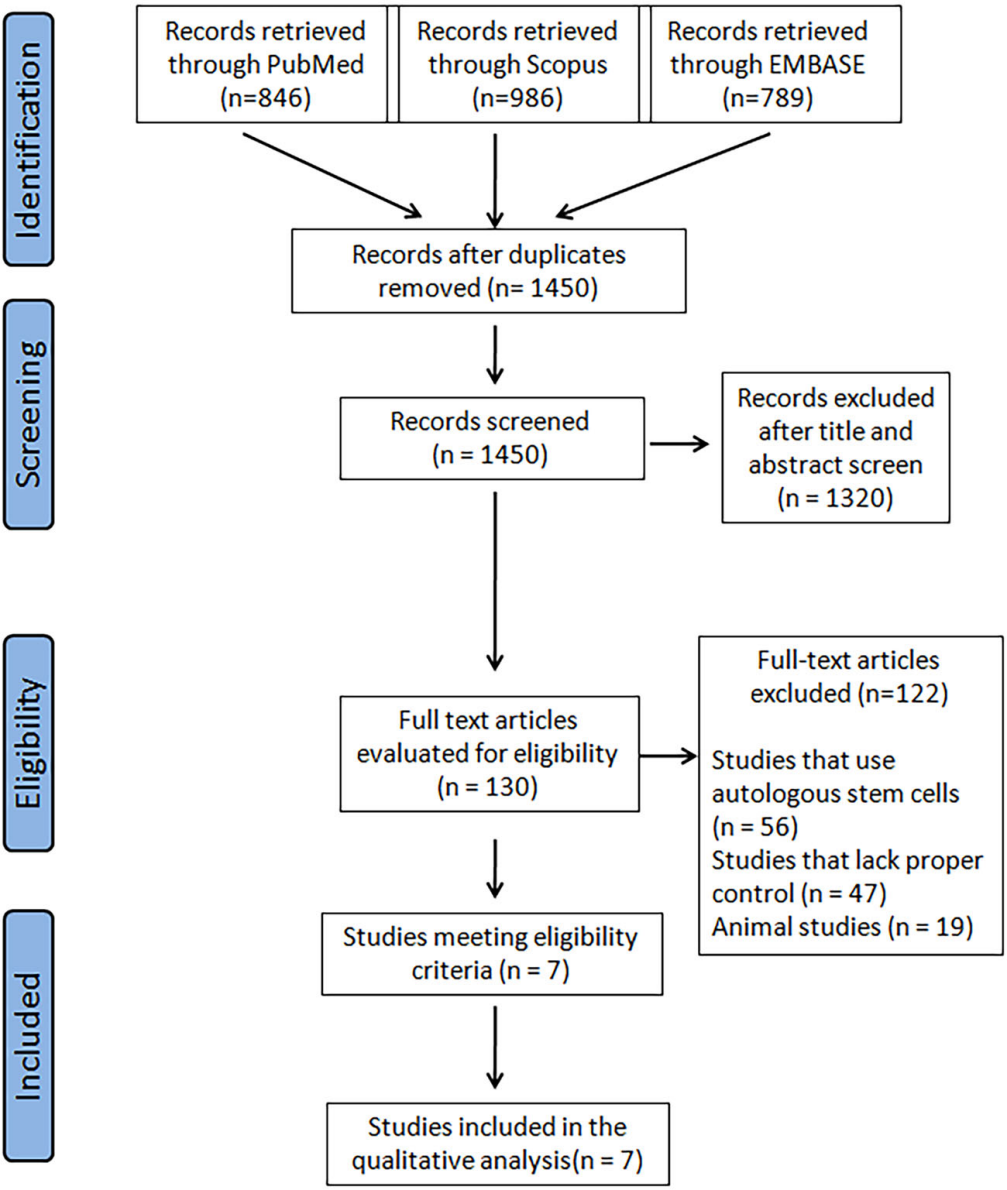
Prisma Flowchart outlining the process of studies selection for review.

The PRISMA flowchart is illustrated in Figure 1. Initially, 1450 articles were identified after the removal of duplicates. After evaluation for eligibility based on inclusion and exclusion criteria, seven articles were finally enroled for analysis. Gupta et al. [11] included two treatment groups, with 25 million and 75 million cells per intervention, and are henceforth represented as separate cohorts as “Gupta 2016 (25 M cells)” and “Gupta 2016 (75 M cells)” respectively for analyses.

### Quality assessment

Of the seven studies included in this review (Table 1), Wang et al. [30], scored 7 on the modified Jadad scale, due to a lack of description of adverse events while the rest of the studies scored 8.

**Table 1:** Quality assessment of the papers included in this study, scored using the modified Jadad scale.

Study	LoE	Study design	QoE/Total
Gupta et al. [11]	1	RCT	MJS 8/8
Koh et al. [14]^a^	2	Prospective comparative study^a^	MINORS 14/16
Vega et al. [29]	1	RCT	MJS 8/8
Gupta et al. [11]	1	RCT	MJS 8/8
Sadri et al. [24]	1	RCT	MJS 8/8
Wang et al. [30]	1	RCT	MJS 7/8
Qiao et al. [22]	1	RCT	MJS 8/8

Abbreviations: LoE, level of evidence; MINORS, Methodological Index for Non‐Randomised Studies; MJS modified Jadad Scale; RCT, randomised controlled trials.

a Koh 2014 was included given that the two groups of patients were indeed randomised, though the patients were not blinded to the intervention received

### Features of included studies

A total of 356 patients with knee OA were included in this review (Table 2). Allogenic MSCs were implanted in 177 patients. There were 111 males and 245 females, with a mean age of 54.2 years. The average follow‐up period was between 12 and 24 months in these studies. Outcome measures were heterogeneous among the articles. These measures included safety data as reported by adverse events, the Western Ontario and McMaster Universities Osteoarthritis Index (WOMAC), the Visual Analogue Scale (VAS) for pain, radiographic magnetic resonance imaging (MRI), and several others.

**Table 2:** Demographics and characteristics of included studies.

Author	Year	Number of patients		Mean age (year) (SD)		BMI (kg/m^2^) (SD)		Stem cell origin	Number of cells (10^6^)
		Intervention (M/F)	Control group (M/F)	Intervention	Control	Intervention	Control		
Gupta 2016 (25 M)	2016	3/7	0/10	58.10 (8.23)	54.90 (8.27)	29.73 (6.09)	28.84 (4.91)	Bone marrow	25/50^a^
Gupta 2016 (75 M)	2016	2/8	3/7	55.00 (6.72)	56.70 (5.19)	28.38 (2.38)	26.40 (3.99)	Bone marrow	75/150^a^
Vega 2015	2015	5/10	6/9	57.3 (9.1)	56.6 (9.2)	NIL	NIL	Bone marrow	40
Gupta 2023	2023	26/47	22/51	51.6 (6.77)	53.6 (6.78)	26.5 (2.58)	26.2 (2.99)	Bone marrow	25
Koh 2014	2014	5/16	6/17	54.2 (2.9)	52.3 (4.9)	25.7 (2.9)	24.7 (3.3)	Adipose	4.11
Sadri 2023	2023	2/18	2/18	52.85 (7.25)	56.1 (7.21)	28.37 (3.26)	29.12 (4)	Adipose	100
Wong 2016	2016	10/8	11/7	54.28	52.37	28.31	27.31	Human umbilical cord	20–30
Qiao 2020	2020	3/7	5/5	62.0 (8.33)	59.7 (7.12)	26.0 (2.86)	26.4 (2.14)	Adipose	50

Abbreviation: SD, standard deviation.

a Gupta 2016 had two separate intervention groups with different cell counts within the same study.

### Clinical outcomes

**Figure 2:**
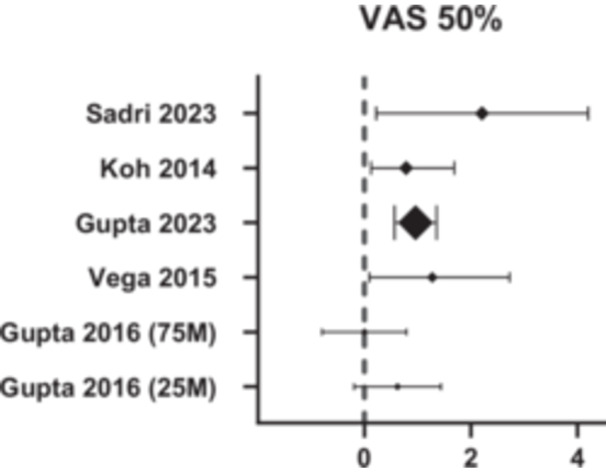
Forest plot showing the efficacy of allogenic MSCs across the studies, with a 50% improvement in VAS score compared to pre‐operation as the threshold. MSCs, mesenchymal stem cells; VAS, Visual Analogue Scale.

Visual analogue scale (VAS) (0–100) is commonly used to assess pain and is used in five of the seven studies included in this review. This includes two arms from Gupta 2016, but only five distinct studies in total. There is a mean improvement in VAS score from 59.45 preoperatively to 28.02 at 12 months follow‐up, representing a 30.4 point reduction in pain score (Figure 2) (Table 3). In comparison, a meta‐analysis on acute pain across various clinical conditions found the minimum clinically important difference (MCID) for VAS score to be between 8 and 40 points [18]. More specifically, another review looking at non‐surgical interventions in knee OA found the MCID for VAS score to be between 10 and 19.0 [5]. Figure 2 also shows a high likelihood of 50% improvement in VAS score from baseline in most studies at 12 months post‐procedure.

**Table 3:** VAS score across the different studies, comparing pre‐ and post‐op, with an odds ratio (95%CI).

Author	Intervention VAS			Control VAS				Odds ratio (50% improvement from baseline)
	Pre‐op	6 Months	12 Months	Regimen	Pre‐op	6 Months	12 Months	
Gupta 2023 (25 M cells^a^)	60.9 (19.7)	24.4	20.6 (±17)	15 mL plasmalyte	61 (23.8)	45.3	39.7(±30)	4.18 (0.64–27.37)
Gupta 2023 (75 M cells^a^)	57.4 (29.0)	37.1	38.3 (±28.3)	15 mL plasmalyte	65.3 (12.2)	43.4	40.5 (±19.4)	1 (0.16–6.16)
Vega 2015	54 (7)	‐	33 (6)	60 mg HA	64 (7)	‐	51(8)	18.91 (0.67–531.61)
Gupta 2023	66.1 (13.96)	40.3	33.5 (17.34)	1 mL plasmalyte, 2 ml HA	65.0 (13.09)	52.5	61.1 (21.85)	9.11 (3.68–22.54)
Yong Gon Koh 2014^b^	44.3 (5.7)		10.2 ± 5.7*	3 mL PRP	45.4 (7.1)		16.2 ± 4.6*	6.04 (0.75–48.87)
Sadri 2023	74.0 ±13.5	31.5 ± 18.7	32.5 ± 15.8	5 mL NS	77.3 ± 114	70 ± 18.5	74.7 ± 15.4	162.46 (1.67, 15829.3)

Abbreviations: CI, confidence interval; VAS, Visual Analogue Scale.

a 25 M refers to the intervention with a 25 million cell count. Data within the same study has been separated given different intervention dosages.

b Yong Gon Koh 2014: Only 24 months of follow‐up data are available.

**Figure 3:**
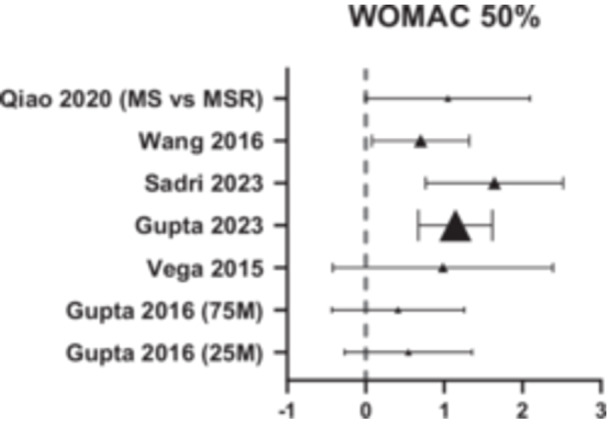
Forest plot showing efficacy of allogenic MSCs across the studies, with 50% improvement in WOMAC score compared to pre‐operation as the threshold. MSCs, mesenchymal stem cells; WOMAC, Western Ontario and McMaster Universities Osteoarthritis Index.

Similarly, the WOMAC score decreased from a mean of 66.3–26.3 at 12 months follow‐up, a 40‐point reduction (Figure 3) (Table 4). Escobar et al. [8] reported an MICD score of 15 for WOMAC in patients who underwent total knee replacement for knee OA [9]. Hence, a reduction of 40 points would mean a significant improvement in the domains of pain, stiffness and daily function who received allogeneic MSCs treatment. Supporting this, in Figure 3, most studies show a high likelihood of 50% improvement in WOMAC score from baseline in most studies at 12 months follow‐up.

**Table 4:** WOMAC score across the different studies, comparing pre‐ and post‐op, with an odds ratio (95%CI).

Author	Intervention WOMAC			Control WOMAC				Odds ratio (50% improvement from baseline)
	Pre‐op	6 Months	12 Months	Regimen	Pre‐op	6 Months	12 Months	
Gupta 2016 (25 M cells)	1270 (425)	750 (605)	552.2 (503.8)	15 mL plasmalyte	1250 (500)	1000 (600)	1016.2 (641.9)	3.5 (0.54, 22.76)
Gupta 2016 (75 M cells)	1480 (480)	1010 (512)	850 (573)	15 mL plasmalyte	1410 (560)	990(504)	1050 (450)	2.58 (0.37, 18.15)
Vega 2015	41(3)	‐	28(5)	60 mg HA	45 (3)	‐	41(6)	9.66 (0.38, 243.69)
Gupta 2023	1412.0 (336.6)	870.6 (297.9)	741.3 (346.1)	1 mL plasmalyte, 2 mL HA	1361.4 (305.2)	1187.7 (469.69)	1363.5 (488.62)	13.92 (4.69, 41.27)
Sadri 2023	58.35 ± 13.25	16.75 ± 13.81	19.05 ± 14.12	5 mL NS	65.42 ± 14.63	55.63 ± 23.17	63.47 ± 20.68	43.89 (5.83, 330.48)
Wang 2016	99.25 ± 31.85	43.13 ± 18.50		3 mL HA	99.377 ± 29.89	71.25 ± 33.29		5.04 (1.21, 20.98)
Qiao 2020	45.8 ± 10.2	30.7 ± 4.18	24.8 ± 4.80	Arthroscopic microfracture, HA	40.9 ± 6.26	27.5 ± 4.3	29.5 ± 2.2	11.17(1, 124.78)

Abbreviations: CI, confidence interval; HA, hyaluronic acid; WOMAC, Western Ontario and McMaster Universities Osteoarthritis Index.

In addition, when compared to the gold standard of treatment, total knee arthroplasty [8], the MCID for pain improvement is 27.1–32.6 (95% CI) (*n* = 209), while that for functional improvement is 28.3–33.9 (95% CI) (*n* = 221). Looking at these MCID values for pain and WOMAC in total knee arthroplasty, allogenic stem cell treatment can achieve similar clinically significant improvements in knee pain and functional outcomes while avoiding the risks and comorbidities of surgery. This indicates that allogenic stem cell treatment could be a viable treatment option for knee OA.

### Adverse Events

Adverse events data was published in four studies [10, 11, 24, 29]. The most common side effects were local reactions to the injection, such as implantation site pain (20%–47%), or articular swelling (10%–53%). However, the complication rates were similar between the controls and the allogenic stem cell‐treated group. Overall, all the studies published concluded that the adverse events were largely self‐limiting and did not differ significantly between the control and treatment groups.

### MRI studies

The efficacy of allogeneic MSCs in knee OA is supported by imaging studies. Three of the papers demonstrated improvement in cartilage volume and quality: a decrease in poor cartilage area quantified by T2 relaxation measurements, associated with an increase in cartilage thickness in the tibia and femoral compartments. The follow‐up time was between 6 and 12 months post‐procedure [22, 24, 29]. Sadri et al. [24] reported a significant increase in cartilage thickness of the tibia medial anterior (baseline1.86 ± 0.53 to 12 months 1.98 ± 0.56; *p* < 0.05) and tibia medial posterior segments (baseline 2.01 ± 0.29 to 12‐month 2.07 ± 0.26; *p* < 0.01). Vega et al. [29] and Sadri et al. [24] reported a correlation between radiological improvement with both WOMAC and VAS improvement whereas Qiao et al. [22] only reported a correlation with WOMAC improvement. Gupta et al. [11] also showed an increase in deep cartilage in the medial tibiofemoral compartment but was not statistically significant. Overall, the MRI studies showed that allogenic MSC treatment has the propensity to improve cartilage thickness in the joint space, and this beneficial effect is sustained up to at least 12 months post‐procedure.

### Histological stain

In terms of histological staining, Qiao et al. [22] showed biopsy specimens from the medial femoral condyle having significant improvement in surface architecture (*p* < 0.001) and increased chondrocyte clustering (*p* = 0.07) [22]. The histological ICRSII scores also improved significantly in four parameters: surface architecture, and surface, mid and overall assessments, with the overall assessment scores improving from 32.0 ± 21.6 at baseline to 55.9 ± 23.2 at 6 months (*p* < 0.05).

## DISCUSSION

This systematic review sieved through multiple databases for literature, using manual review and snowballing to add to the depth of our literature search. It covered six RCTs and one prospective study with randomised patient groups, which included 356 patients and compared the efficacy of MSCs intra‐articular injection and control injection.

The results showed that MSC therapy was safe and well tolerated and can significantly reduce postoperative pain in OA patients.

Current non‐surgical treatment of knee OA includes the use of oral non‐steroidal anti‐inflammatory drugs (NSAIDs), intra‐articular NSAIDs and intra‐articular hyaluronic acid [13, 15]. A study by Hmamouchi et al. showed that MCID for oral NASIDs in knee OA is a 16% reduction from baseline in WOMAC score [12]. In our current review, the cut‐off for the effectiveness of therapy was chosen as 50% improvement from baseline, which is at the upper limit of MCID published by most studies. For example, a recent review by Concoff et al. showed that various non‐surgical treatments have a huge range of MCID values, from 20% to 50% improvement in WOMAC and VAS scores [5]. Despite the stringent criteria of 50% improvement from baseline, the calculated odds ratio showed that most of the studies in this review were effective in the treatment of knee OA. Thus, allogeneic stem cell therapy could be a viable alternative to conventional non‐surgical treatment options for knee OA.

### Advantages of allogeneic MSCs

First, allogeneic MSCs derived from adipose tissue and bone marrow offer logistical convenience [22]. While autologous MSCs require several weeks for isolation, in‐vitro expansion and release, this might be circumvented by allogenic MSCs' potential for off‐the‐shelf availability [2]. Notably, the scalability and widespread availability of allogeneic MSCs streamline manufacturing processes and facilitate their integration into clinical practice.

Furthermore, allogenic MSCs allow for optimal donor selection. Inter‐patient differences in gender, BMI, site, age and diseases introduce variation to the MSCs extracted from autologous sources [7, 27]. Advanced age, co‐morbidities and availability of tissue at the time of treatment may constrain the extraction of MSCs from autologous sources. Allogenic sources allow for optimal patient selection and offer the option of pooling multiple donors, potentially reducing variation in the quality of MSCs injected to achieve a more stable profile [31].

### Potential downsides of MSCs

Firstly, concerns have been raised about the genetic stability of MSCs [20], including the possibilities of senescence [3, 28], chondrocyte phenotype instability and tumorigenesis following MSC implantation. However, no studies have shown evidence of malignant transformation after MSC implantation to the knee [26, 27]. Additionally, comorbidities such as diabetes mellitus, obesity, and metabolic syndrome may influence the differentiation of MSCs, potentially leading to adipogenesis rather than chondrogenesis. Lastly, repeated administration of MSCs may provoke an immunological response, resulting in the production of alloantibodies [4].

### Limitations

Limitations of our study include: (1) Limited sample size of clinical trials. (2) Varying follow‐up periods among the studies. (3) Baseline characteristics and comorbidities of patients amongst studies. (4) Potential bias in published studies. First, the limited sample size of clinical trials included in this review poses a notable constraint. While efforts were made to encompass all available literature, the restricted pool of eligible studies may affect the generalisability of findings and the robustness of statistical analyses. Second, the absence of long‐term data beyond the two‐year follow‐up timeframe across all studies restricts our ability to assess the enduring efficacy and safety profile of allogeneic MSC therapy in knee OA. Future research with extended follow‐up periods is imperative to elucidate the sustainability of treatment outcomes.

Furthermore, the variability in patient characteristics across included studies introduces complexity. Variations in demographic profiles, disease severity, comorbidities and prior treatments among participants may influence treatment responses and confound interpretation. Although subgroup analyses were conducted where feasible, the heterogeneity in patient populations remains a potential source of bias.

Lastly, potential bias in published studies constitutes a notable limitation. Publication bias toward studies reporting positive outcomes may skew the overall perception of allogeneic MSC therapy in knee OA. Efforts were made to mitigate this bias through comprehensive literature searches and inclusion criteria; however, the possibility of selective reporting cannot be entirely discounted.

## CONCLUSION

According to current literature, allogenic MSC implantation into patients with knee OA provides sustained clinical improvements in pain and function up to at least 12 months post‐procedure. These results are supported by both imaging and histological outcomes. The safety profile of allogeneic MSCs is excellent as well, with minimal adverse events mainly limited to local reaction to injection and no long‐term adverse effects.

## AUTHOR CONTRIBUTIONS

**Randy Loke You Jie**: Methodology; data curation and analysis; writing—original draft preparation. **Zachary Chu**: Data curation and analysis; writing—original draft preparation. **Jonathan Liang**: Data curation and analysis; writing—original draft preparation. **Don Koh**, **Soong Junwei**, **Lee Kong Hwee**: Manuscript editing. **Hamid Rahmatullah Bin Abd Razak**: Conceptualisation; supervision.

## CONFLICT OF INTEREST STATEMENT

The authors declare no conflicts of interest.

## ETHICS STATEMENT

None declared.

## Data Availability

Data sharing is not applicable to this article as no data sets were generated or analysed during the current study.
